# Treatment of Metastatic Primary Extramammary Paget Disease With Combination Anlotinib and Tislelizumab: A Case Report and Review of the Literature

**DOI:** 10.3389/fmed.2022.891958

**Published:** 2022-05-24

**Authors:** Xin Yin, Xiaoqing Li, Muli Li, Qing She, Yan Liu, Xiaodan Chen, Suhua Ma, Qian Ma, Zhangkan Huang, Lin Xu, Xiaozhun Huang, Zhengyin Zhan, Xu Che

**Affiliations:** ^1^Department of Hepatobiliary Surgery, National Cancer Center, National Clinical Research Center for Cancer, Cancer Hospital and Shenzhen Hospital, Chinese Academy of Medical Sciences and Peking Union Medical College, Shenzhen, China; ^2^Department of Pathology, National Cancer Center, National Clinical Research Center for Cancer, Cancer Hospital and Shenzhen Hospital, Chinese Academy of Medical Sciences and Peking Union Medical College, Shenzhen, China

**Keywords:** extramammary Paget disease, anlotinib, tislelizumab, neuroendocrine differentiation, survival

## Abstract

Extramammary Paget’s disease (EMPD) is a rare cutaneous neoplasm with distant metastases and a poor prognosis. We report the case of a 63-year-old male patient exhibiting stage IV primary EMPD with neuroendocrine differentiation, and harboring a somatic mutation in AMER1. After four cycles of Anlotinib combined with Tislelizumab, the patient achieved partial response for the metastatic lesions according to mRECIST1.1 criteria. Total positron emission tomography and computed tomography (PET-CT) scans revealed a significant reduction in SUV from 18.9 to 5.3, and the serum CEA decreased to normal levels after the treatment regimen. However, the patient developed fractures of the fourth and fifth thoracic vertebrae during the treatment. Therefore, percutaneous vertebroplasty was performed, and the patient experienced severe postoperative pneumonia and died from pulmonary encephalopathy and respiratory failure in June 2021. The overall and progression-free survival of the patient after diagnosis were 9 and 8 months, respectively. During the systemic treatment, the patient suffered grade 1 rash in the back and thigh and grade 1 hypertension. Nevertheless, the combination treatment of anlotinib and tislelizumab had a favorable clinical outcome and provided a survival advantage, and should be considered a therapeutic option for patients with AMER1-mutant metastatic EMPD.

## Introduction

Extramammary Paget’s disease (EMPD) is a rare cutaneous neoplasm that accounts for 6.5% of all Paget’s disease cases (1). Most EMPD cases are diagnosed as carcinoma *in situ*, which usually shows indolent disease progression. However, once Paget cells invade deeply into the dermis, they frequently metastasize to the regional lymph nodes (LN) and distant organs (2). Distant metastasis is associated with poor prognosis and <10% 5-year survival rate due to the limited efficacy of conventional chemotherapies (1, 3).

Recent studies have elucidated the pathways underlying EMPD development and progression, which can lead to the development of novel treatment approaches for metastatic EMPD. For instance, genomic analyses of EMPD lesions have identified somatic mutations in various genes, including TP53, ERBB, NRAS, BRAF, PIK3CA, and AKT1 (4, 5). This strongly suggests that the pathways downstream of ERBB2/HER2, such as the RAS/RAF-MEK-ERK and PI3K-AKT-mTOR pathways, may drive EMPD progression (5), and should be explored further as potential therapeutic targets for metastatic EMPD. Patients with metastatic EMPD may be considered for chemotherapy, targeted therapy, or immune checkpoint inhibitors. The tyrosine kinase inhibitor Anlotinib (6) has multiple targets and exerts its anti-tumor effect by inhibiting PI3K/AKT phosphorylation (7). Combined targeted therapy and immune checkpoint inhibitors may be appropriate for patients without good performance status.

Herein, we report a case of metastatic EMPD that responded to the combination therapy of Anlotinib and the checkpoint inhibitor Tislelizumab.

## Case Presentation

**FIGURE 1:**
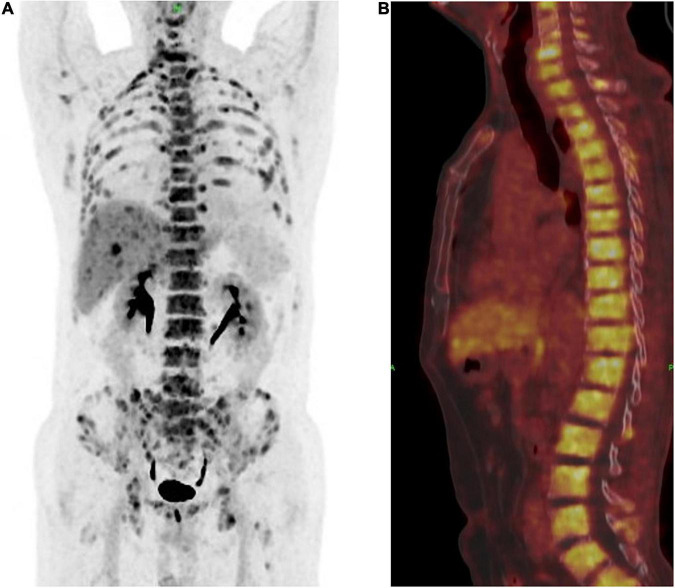
**(A)** Positron emission tomography and computed tomography (PET-CT) showed a multiple bone destruction with bone metastases including whole vertebra, occipital bone, ribs of double side, appendicular skeleton, and pelvis. **(B)** PET-CT showed a whole vertebra metastatic Primary EMPD, showing that a most obvious SUV of 18.9.

**FIGURE 2:**
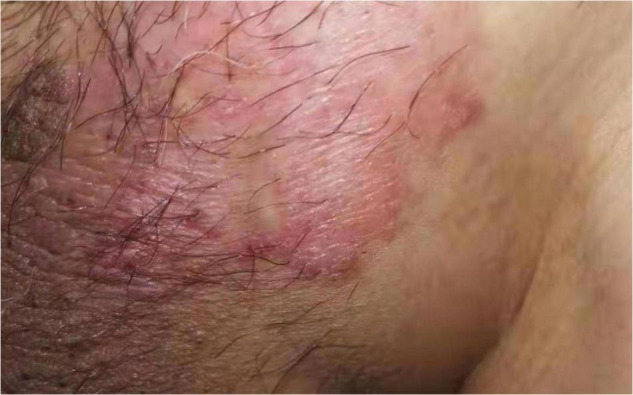
The perineal lesions were about 4 cm × 3 cm in size, with obvious rash, erythema and erosion, accompanied by pruritus, no pain and exudation.

**FIGURE 3:**
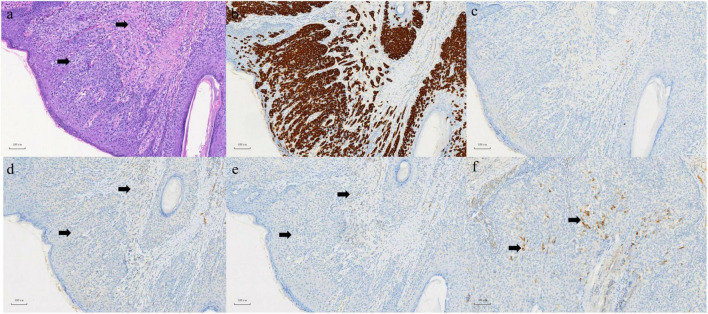
**(a)** In epidermis and dermis, there are abundant pale-staining cytoplasmic, which are flaky, small nests or scattered cells in HE staining, involving both the dermis and skin appendages. Left arrow: Paget cell. Right arrow: infiltrating adenocarcinoma. **(b)** Positive expression of CK (7) in EMPD section 100×. **(c)** Negative expression of CK (20) in EMPD section 100×. **(d)** Negative expression of CD (56) in EMPD section 100×. **(e)** Negative expression of ChrA in EMPD section 100×. **(f)** Focal positive expression of synaptophysin in EMPD section 100×.

A 63-year-old male who was diagnosed with eczema of the scrotum in 2012 and did not pursue treatment visited our hospital on 12th October, 2020 on account of back pain. Positron emission tomography computed tomography (PET-CT) showed erosion and metastatic nodules in the occipital bone, entire vertebra, bilateral ribs, appendicular skeleton and the pelvic bone (Figures 1A,B). The patient was also positive for hepatitis B, and the serum levels of CA19.9, AFP, NSE, and CEA were 43.24 U/ml, 2.90 ng/ml, 15.62 ng/L, and 1,488 ng/ml, respectively. Fine-needle aspiration cytology (FNAC) was performed on the hepatic metastatic node and right groin lymph node, and the biopsies indicated neuroendocrine differentiation. However, based on the clinical presentation and case history, we suspected that the primary tumor was eczema of scrotum (Figure 2). Perineal skin mass resection surgery was subsequently performed, and histological examination of the epidermis and dermis revealed lightly stained tumor cells with dense cytoplasm that were arranged in the form of sheets, small nests or scattered masses. These histological features were consistent with Paget’s disease with infiltrating adenocarcinoma. The tumor grew 0.4 cm deep into the dermis and involved the skin appendages. Immunohistochemical staining indicated that the EMPD tumor cells were positive for GATA3 (3+), CK7 (3+), GCDFP15 (1+), androgen receptor (2+), HER2 (2+), ER (1+), CEA (3+), and synaptophysin (focal+), and negative for CK20, P16, S-100, HMB-45, P40, PR, CD56, and ChrA. However, the FISH test for HER2 was negative (Figure 3). Based on the histopathological and immunohistochemical findings, we diagnosed the case as cT1N2M1 primary EMPD with neuroendocrine differentiation on 20th October, 2020.

Sequencing analysis of the biopsied specimens revealed a mutation in AMER1 exon 2 (c.1724G > A, p.R575Q) (Supplementary Figure 1), which has been identified as the causative mutation of aberrant PI3K/AKT/mTOR signaling in gastric cancer cells, and promotes the progression and metastasis of gastric cancer (8). In addition, the patient had a tumor mutational burden (TMB) of 2.38 mut/Mb and a stable microsatellite status (MSS), along with low expression of programmed death-ligand 1 (PD-L1). Given the extensive bone metastases, especially in the entire vertebra, we did not consider conventional chemotherapy due to its myelosuppressive effects. Based on the poor physical condition of the patient and genetic results, we initiated a regimen of Anlotinib (12 mg) in combination with the IgG4 anti-PD-1 humanized monoclonal antibody Tislelizumab (9, 10) after a multi-disciplinary panel discussion.

**FIGURE 4:**
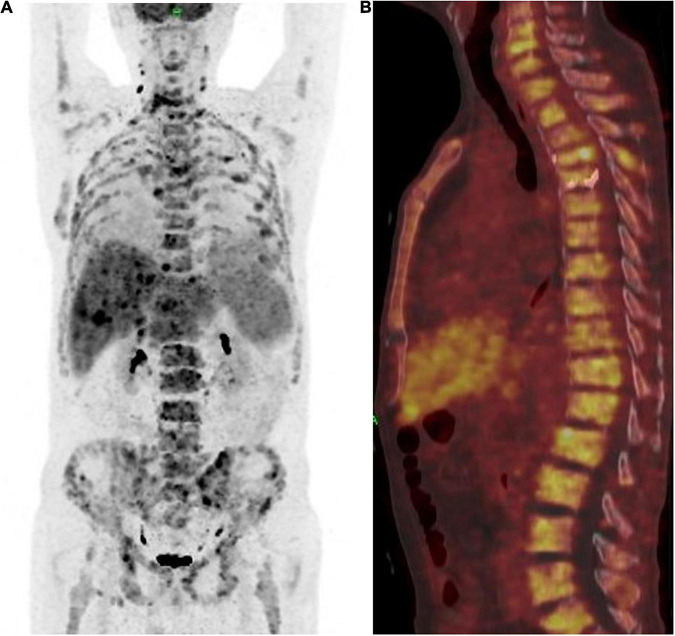
**(A)** Positron emission tomography and computed tomography showed the patient achieved partial response (PR) according to the mRECIST1.1 on metastatic lesions. **(B)** PET-CT showed a whole vertebra metastatic Primary EMPD, showing that a most obvious decrease of SUV from 18.9 to 5.3.

The patient achieved partial response (PR) for the metastatic lesions according to the mRECIST1.1 criteria after four cycles of treatment. The total PET-CT scan (Figures 4A,B), showed an obvious decrease in SUV from 18.9 to 5.3, and the serum level of CEA also dropped within the normal range. Computed tomography (CT) and magnetic resonance imaging (MRI) showed that both hepatic and lymph node (regional and distant) metastases achieved stable disease (SD) status as per RECIST1.1. However, the patient developed fractures in the fourth and fifth thoracic vertebrae during the treatment period due to an accident. Therefore, the treatment regimen was suspended for 4 weeks and percutaneous vertebroplasty was performed in that interval. External thoraco-lumbosacral orthosis was used postoperatively.

The patient experienced severe postoperative pneumonia. Chest CT showed that the metastatic lymph nodes of thoracic vertebrae and mediastinum were stable. He died from pulmonary encephalopathy and respiratory failure in June, 2021 with overall survival (OS) of 9 months and progression-free survival (PFS) of 8 months. During the treatment regimen, the patient experienced grade 1 rashes on the back and thigh, as well as grade 1 hypertension.

## Discussion

Chemotherapy remains the cornerstone of EMPD management. However, there is currently no standardized treatment for metastasis (11, 12). Kato et al. retrospectively analyzed 17 EMPD patients with multiple metastases, of which eight were treated with 5-fluoruuracil (FU)/cisplatin and nine patients chose best supportive care (13). The median PFS (mPFS) and median OS (mOS) of the 5-FU/cisplatin group were 6.2 and 19.4 months, respectively, and were not significantly longer than that of the supportive care group (13). Tokuda et al. retrospectively examined 22 patients with advanced EMPD who received 5-FU/cisplatin therapy, and found that mPFS and mOS were 5.2 and 12 months, respectively (14). Yoshino et al. conducted a multicenter, retrospective study to evaluate the efficacy of docetaxel as a first line chemotherapy for 13 metastatic EMPD patients, and found that the mPFS and mOS were 7.1 and 16.6 months, respectively (15). Docetaxel and low-dose 5-FU/cisplatin (FP) have been used to treat locally advanced EMPD, although data is limited. While docetaxel monotherapy can result in grade 3 or 4 myelosuppressive events, FP therapy usually requires repeated hospitalization (15). Although conventional chemotherapies have been used to treat distant metastases for a long time, some studies indicated possible efficacy of chemotherapies, however, no prospective study has shown that conventional chemotherapy improves overall survival. Guercio et al. reported a case of metastatic EMPD with treatment response to checkpoint inhibitor immunotherapy (16). The patient had diffuse metastasis and had previously progressed after receiving chemotherapy and alpelisib, an oral α-specific PIK3CA inhibitor. The treatment was discontinued due to the toxic effects of alpelisib such as nausea, grade 3 rash and hyperglycemia, as well as disease progression. The new treatment regimen consisting of four cycles of 1 mg/kg ipilimumab plus 3 mg/kg nivolumab resulted in a durable partial response lasting 7 months (16).

Evidence-Based Clinical Practice Guidelines recommends chemotherapy, targeted therapy or immune checkpoint inhibitors for patients with metastatic EMPD (11). Around 15–58% of EMPD patients overexpress HER2, which likely plays a crucial role in the development and progression of EMPD (17). Multiple cases of HER2-positive advanced EMPD have been reported that respond to the anti-HER2 antibody trastuzumab, either as a monotherapy or combined with other chemotherapies (18). HER2 lies upstream of the RAS–RAF–MEK–ERK and PI3K–AKT–mTOR pathways, which accelerates cell growth and promotes survival (19). Furthermore, DNA sequencing of EMPD tumors has shown that upregulation of HER2 and its downstream effectors is associated with the progression of EMPD (3). However, our patient was HER negative and harbored a mutation in the AMER1 exon 2 (c.1724G > A, p.R575Q). AMER1 inhibits the PI3K/AKT/mTOR pathway by blocking PI3K phosphorylation (8). Unfortunately, we did not have access to the PI3K specific inhibitor alpelisib. It had been demonstrated that treatment with alpelisib prolonged DFS among patients with PIK3CA-mutated, HER2-negative advanced breast cancer who had received endocrine therapy previously (20). The tyrosine kinase inhibitor Anlotinib (6) has multiple targets and exerts its anti-tumor effect by inhibiting PI3K/Akt phosphorylation (7). In a recent study, Anlotinib mainly inhibited angiogenesis and tissue tumor cell migration by inhibiting c-Kit, VEGFR2, VEGFR3, FGFR1-4, PDGFR α and β, and other targets, thus inhibiting tumor by inhibited PI3K/AKT/Bad phosphorylation and promoted apoptosis of tumor cells by activating RAS protein expression (21, 22). In the present study, the combination of Anlotinib and Tislelizumab significantly prolonged the DFS of the patient to 8 months.

Tislelizumab was an investigational humanized IgG4 monoclonal antibody and engineered to minimize binding to FcγR on macrophages in order to limit antibody-dependent phagocytosis, a potential mechanism of resistance to anti-PD-1 therapy. Dose-escalation and dose-expansion study had assessed the safety, tolerability, pharmacology and clinical activity of tislelizumab in patients with advanced solid tumors (10). Previous studies have reported low rates (10%) of PD-L1 expression in EMPD tumor cells (23–25), and checkpoint inhibitor blockade is recommended for patients with mismatch repair or microsatellite instability, or high mutational burden (11), that does not mean that there would be poor anti-tumor effect from checkpoint inhibitors in all EMPD cases. A prior study identified germline mismatch repair gene missense mutations in 8 of 20 EMPD tumors, with a proportion of cases exhibiting microsatellite instability (MSI), lending rationale for consideration of checkpoint inhibitor immunotherapy in such cases (26). Our patient responded to immune checkpoint blockade despite microsatellite stability. Although PD-L1 expression correlates with the response to PD-1/PD-L1 targeted therapies in some settings, it often fails to predict treatment response. Furthermore, there are currently no established criteria for high versus low TMB in EMPD. One study reported successful outcome of immune checkpoint inhibition for metastatic EMPD with low PD-L1 expression, microsatellite stability and TMB of 4.4 mut/MB (16). Likewise, our study also provides preliminary evidence for extending the indication for immunotherapy to primary metastatic EMPD.

There is no report at present of primary metastatic EMPD with neuroendocrine differentiation. Furthermore, only one case of canal adenocarcinoma with neuroendocrine features accompanying secondary EMPD has been reported in an elderly male patient, who responded to 5-FU, leucovorin and oxaliplatin (FOLFOX6) (27). The present case was that of a rare primary EMPD with neuroendocrine features, which indicated poor prognosis on account of metastases. For advanced malignancies lacking standard treatment, therapy based on mutated genes and pathways may improve prognosis. While our findings support further investigation into the safety and efficacy of targeted therapy combined with immune checkpoint inhibitors for metastatic EMPD, achieving more than 8 months of DFS and 9 months of OS in the poor performance status patient. Tislelizumab concomitant with Anlotinib has tolerable toxicity and favorable antitumor activity in patients with previously treated advanced tumors (28), no additional toxicity was observed. These findings contribute new, important information on Anlotinib plus Tislelizumab in metastatic EMPD, particularly demonstrated the Anlotinib inhibit the PI3K/AKT signaling pathway in the EMPD with AMER1 mutations, provided evidences and a basic principle for using Anlotinib plus Tislelizumab to treat patients with advanced malignancy who lack effective treatment choices.

In conclusion, an AMER1-mutant patient with rare primary EMPD presenting neuroendocrine features responded to the combination treatment of Anlotinib and Tislelizumab, which suggest an effective and tolerable therapeutic option for this rare disease.

## Author Contributions

LX, XL, ML, and QS contributed to conception and design of the study. YL, XC, SM, QM, ZH, ZZ, and XC contributed to the acquisition, analysis, or interpretation of data for the work. XY and XH wrote the first draft of the manuscript. All authors contributed to the article and approved the submitted version.

## Conflict of Interest

The authors declare that the research was conducted in the absence of any commercial or financial relationships that could be construed as a potential conflict of interest.

## Publisher’s Note

All claims expressed in this article are solely those of the authors and do not necessarily represent those of their affiliated organizations, or those of the publisher, the editors and the reviewers. Any product that may be evaluated in this article, or claim that may be made by its manufacturer, is not guaranteed or endorsed by the publisher.

## Supplementary Material

Supplementary Figure 1 The IGV picture of AMER1 exon 2 (c.1724G > A, p.R575Q).
