# Supplementary figures and images for: Treatment of Metastatic Primary Extramammary Paget Disease With Combination Anlotinib and Tislelizumab: A Case Report and Review of the Literature
Source: Front Med (Lausanne). 2022 May 24;9:891958. doi: 10.3389/fmed.2022.891958 (PMC9170962; doi:10.3389/fmed.2022.891958)

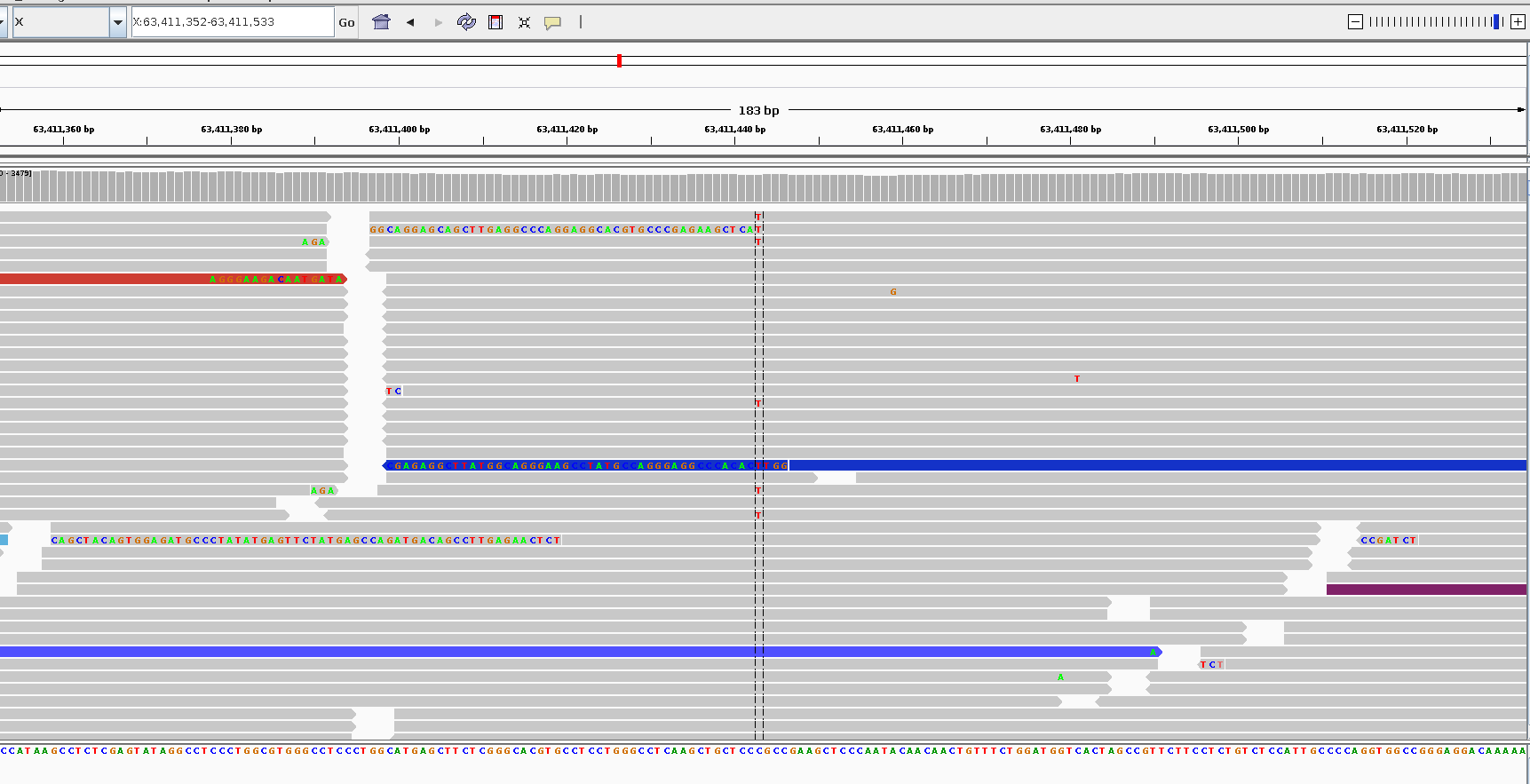

Supplement: Supplementary Figure 1 — The IGV picture of AMER1 exon 2 (c.1724G > A, p.R575Q). [file Image_1.PNG]
